# The soothing function of touch: affective touch reduces feelings of social exclusion

**DOI:** 10.1038/s41598-017-13355-7

**Published:** 2017-10-18

**Authors:** Mariana von Mohr, Louise P. Kirsch, Aikaterini Fotopoulou

**Affiliations:** Research Department of Clinical, Educational and Health Psychology, University College London, London, United Kingdom

## Abstract

The mammalian need for social proximity, attachment and belonging may have an adaptive and evolutionary value in terms of survival and reproductive success. Consequently, ostracism may induce strong negative feelings of social exclusion. Recent studies suggest that slow, affective touch, which is mediated by a separate, specific C tactile neurophysiological system than faster, neutral touch, modulates the perception of physical pain. However, it remains unknown whether slow, affective touch, can also reduce feelings of social exclusion, a form of social pain. Here, we employed a social exclusion paradigm, namely the Cyberball task (N = 84), to examine whether the administration of slow, affective touch may reduce the negative feelings of ostracism induced by the social exclusion manipulations of the Cyberball task. As predicted, the provision of slow-affective, as compared to fast-neutral, touch led to a specific decrease in feelings of social exclusion, beyond general mood effects. These findings point to the soothing function of slow, affective touch, particularly in the context of social separation or rejection, and suggest a specific relation between affective touch and social bonding.

## Introduction

Mammals have a well-recognized need for social proximity and attachment. Consequently, it is not surprising that some of the most distressing life experiences involve the dissolution of social bonds. Long-term isolation, rejection and loneliness have been associated with physical and psychological negative health outcomes^1^. Even small-scale social exclusion (i.e., ostracism) using a computerised ball-tossing game to manipulate social exclusion in an experimental setting (i.e., the Cyberball paradigm^2^) has been found to induce strong negative reactions, including effects in affect, cognition and physiology^3^. Given the importance of social proximity and attachment to survival, threats to social connection could be as harmful to our wellbeing as threats to physical safety, such as pain. Even more, it has been proposed that the physical pain system has been co-opted to signal when social relationships are in threat^4,5^, with neuroimaging evidence suggesting an overlap in brain regions implicated in the affective component of physical pain and ostracism, namely the dorsal anterior cingulate cortex (dACC) and anterior insula^6^ (although see^7,8^). In this sense, ostracism may activate a threat detection system that is experienced as a ‘social pain’ to promote re-connection and social proximity and bonding^4,5^. Indeed, research suggests that social exclusion motivates individuals to seek interpersonal reconnection^9,10^.

Given the above perspectives on ostracism, one may predict that social connection and support may buffer the distressing effects of ostracism. Social support has known beneficial effects on distressing life events^11^ as well as physical health^12,13^. Animal models suggest that the buffering effects of social support include the regulation of stress-related activity in the autonomic nervous system and hypothalamic-pituitary-adrenal (HPA) axis^14^. Similarly, in humans, social supportive behaviours following stress conditions seem to attenuate multiple stress systems, including the autonomic nervous system and HPA-axis (see^13^ for a comprehensive review), possibly mediated by neuropeptides involved in social bonding and affiliative behavior, including oxytocin^15^. Further, neuroimaging studies from neighboring topics indicate that social support reduces activity in brain regions implicated in emotion and homeostatic regulation (i.e., anterior cingulate cortex, dorsolateral and ventrolateral prefrontal cortex)^16,17^. Moreover, cues of social support from a partner reduce physical pain^18,19^. Together, these lines of research suggest that neural and hormonal responses to threat cues are minimized when social support is provided^20^. Consequently, it is likely that social support may also buffer ostracism-related effects.

However, this question has received very little attention in science. Specifically, while self-reported supportive daily life interactions have been shown to diminish neuroendocrine stress responses to social stressors as well as decrease activity in the dACC following ostracism^16^, to date, only two studies have directly examined the buffering effects of social support on ostracism. Specifically, these studies suggest that the presence of a friend in high self-esteem individuals^21^, or supportive versus non-supportive texts^22^, reduce feelings of distress caused by social exclusion. However, as systematically reviewed elsewhere^23^, experimental manipulations of actual or primed supportive social presence have poor explanatory power, as they entail many confounds such as familiarity, attention and social desirability effects. One way through which we can study the effects of social support with greater validity, specificity and experimental control is by focusing on comparable conditions of embodied social support^17,20^ and particularly affective, social touch that conveys social support^24,25^. Specifically, social touch has been associated with communicating different intentions and emotions. For example, anger has been associated with hitting and squeezing, disgust with a pushing motion, whereas sympathy and love have been associated with stroking^24^. Nevertheless, this study did not check for different tactile systems as reviewed below.

Manipulations of affective touch are also theoretically important, as touch seems to have a unique contribution to the formation of social bonds^26,27^. In non-human mammals, tactile stimulation by conspecifics has analgesic and stress-alleviating effects^28^ mediated by neurobiological pathways involved in social bonding^5^. Similar beneficial effects are increasingly studied in humans. For instance, touch-based interventions can improve clinical outcomes in patients with fibromyalgia, rheumatoid arthritis and pre-term infants^29,30^. Furthermore, social touch has been suggested as a stress buffer, playing a critical regulatory role in the body’s responses, including cortisol and heart rate responses^31^, to acute life stressors, which ultimately promotes social connection^32^. Supportive of this notion, a recent study suggests that touching a teddy bear mitigates feelings of social exclusion to increase pro-social behaviour^33^. Although further research is needed to fully investigate the mechanisms underlying the buffering effects of touch in humans, it has been proposed that social, affective touch works as a potent interpersonal homeostatic regulator, particularly during early development^34^. According to some theorists such social, homeostatic regulation may involve primarily thermoregulatory processes^32,35^.

Recent research has further shown that there are specific C Tactile (CT) afferent fibres that respond selectively to gentle stroking touch, mediated by a specific neurophysiological system^36^. CT afferent fibres are thought to code pleasant tactile sensations, which selectively respond to slow velocities of tactile stimulation (1–10 cm/s)^36–38^. Critically, research suggests a relationship between slow, CT touch and pain. For instance, gentle slow touch, likely activating CT fibres, increases μ-opioid system activity^39^, which is involved in pain regulation and social connection^5^, whereas opioid blockade modulates the perception of pleasantness of slow CT-optimal touch^40^. Further, recent studies on pain suggest that slow CT-optimal touch modulates subjective^41^ and neural responses to noxious stimulation^42^. However, it remains unknown, whether slow CT-optimal affective touch may affect the distress, or ‘social pain’ associated with ostracism^4^. As mentioned above, this kind of dynamic, slow touch is associated with neurophysiological specificity^38,43^. A recent study suggests that this particular kind of slow dynamic touch, but not the faster stroking touch also tested here as a control condition, conveys specifically positive social intentions such as social support^25^. Thus, we can contrast affective slow touch, that is known to be mediated by the CT system and is typically perceived as pleasant and socially supportive, with faster touch, but otherwise identical touch, that is known not to activate the CT system optimally and is typically judged to feel ‘neutral’ and without a specificity in communicating social intentions. Accordingly, this affective touch manipulation affords experimental control and validity regarding different conditions of social support, while also allowing interpretations of neurophysiological relevance.

Therefore, the present study employed a well-validated paradigm, namely the Cyberball task^2^, to manipulate ostracism in eighty-four healthy females. Following social exclusion, slow affective touch (at CT optimal speeds) was delivered to half of the participants, while fast neutral touch (at non CT optimal speeds) was delivered to the other half. Using these manipulations, we investigated the hypothesis that slow, affective touch would lessen the distress caused by ostracism more than fast, neutral touch.

## Methods

### Participants

Eighty-four females were recruited via the University College London (UCL) Psychology Subject Pool and were compensated for their participation with £8 or 1 credit. The sample size was determined based on prior power calculations (Cohen’s d set at 0.4; G*Power 3.1) in accordance with the average effect sizes reported in experimental social psychology^44^ and other social experimental studies manipulating touch in relation to physical or social pain^33,45^. The UCL ethics committee approved this study and the experiment was conducted in accordance with the Declaration of Helsinki. Only females were recruited to control for gender effects related to touch^27,46^. As presented in Table 1, there were no significant differences between the groups on age, ethnicity, education or any other demographic variable.

**Table 1:** Demographic characteristics for participants allocated to the slow and fast group.

	Slow Touch Group (n = 42)		Fast Touch Group (n = 42)		*t*	*p*
**Age (in years)**	22.21 (2.10)		22.86 (3.06)		−1.12	0.27
**BMI**	20.84 (2.43)		21.44 (3.66)		−0.88	0.38
Missing	2		1			
	**N**	**%**	**N**	**%**	***X*** ^**2**^	***p***
**Relationship Status**					0.43	0.51
In a current relationship	21	50	24	57.1		
Single	21	50	18	42.9		
**Ethnicity**					4.23	0.65
Caucasian	10	23.8	6	14.3		
Asian-British/Asian	23	54.8	29	69		
Mixed/Multi-racial	3	7.1	3	7.1		
Arabic	1	2.4	0	0		
Hispanic/Latino	2	4.8	1	2.4		
Black/ Black British	1	2.4	0	0		
Other	2	4.8	3	6		
**Highest Level of Education Completed**					0.39	0.82
High School	16	38.1	16	38.1		
Bachelor’s Degree	20	47.6	18	42.9		
Master’s Degree	6	14.3	8	19		
**Sexual Orientation**					0.13	0.94
Heterosexual	36	85.7	37	88.1		
Homosexual	1	2.4	1	2.4		
Bisexual	5	11.9	4	9.5		

Age, BMI and mood are presented as mean (standard deviation).

### Design

We employed a 2 (ostracism: inclusion/baseline vs. exclusion; within-subjects factor) × 2 (touch velocity: slow vs. fast; between-subjects factor) mixed design, using the Cyberball paradigm to manipulate ostracism^2^ and randomly assigning participants to a slow touch (n = 42) or, a fast touch group (n = 42) to manipulate affective social support following exclusion. This mixed design, with a between-subjects manipulation of affective touch was judged as necessary, given that the Cyberball paradigm cannot be implemented optimally in repeated measures design (pilot studies indeed revealed that subjects were ‘suspecting’ the rejection/exclusion manipulations when these were repeated). Hence, all participants completed the inclusion/baseline and exclusion conditions. However, as far as our between-group manipulation goes, we needed a baseline measure without any between-group manipulation in order to make sure there were no baseline differences across groups. Thus, our between-group manipulation only took place following the exclusion, but not inclusion/baseline, condition. Our main measure included the Need-threat scale, as well as manipulation checks conducted on affect, the Cyberball task and perceived pleasantness of the touch (see below).

### Procedure and Materials

Upon obtaining written informed consent, participants were told they would be playing an online ball-tossing game against two other participants (who were in fact computer-generated) in order to measure their mental visualization skills^2^. Participants could throw to whomever they wished, and they believed the other “players” could do so as well. Participants’ photographs were taken to maintain the deception. Two adjacent stroking areas, each measuring 9 cm × 4 cm, were then marked on the participant’s left forearm in order to alternate between tactile stimulation sites and minimise habituation^47^.

Participants first completed computerized demographic questionnaires. Participants then played the Cyberball-inclusion game for approximately 2–3 minutes. This corresponded to a 30 ball-tosses game, where all players received equal number of ball-tosses. Upon completion, participants rated twenty-items (e.g., ‘I felt I belonged to the group’, ‘I felt liked’; corresponding to the ‘Need-threat scale’^48^) indexing fundamental needs often threatened by ostracism (i.e., belonging, self-esteem, meaningful existence, control). Participants’ responses were averaged across each subscale to yield an averaged total index of need-threat level (Cronbach α = 0.87), with lower scores indicating greater threat. This was the main self-report measure of the effects of ostracism in this study, as in most studies using this paradigm (e.g.^21,48,49^).

**Figure 1:**
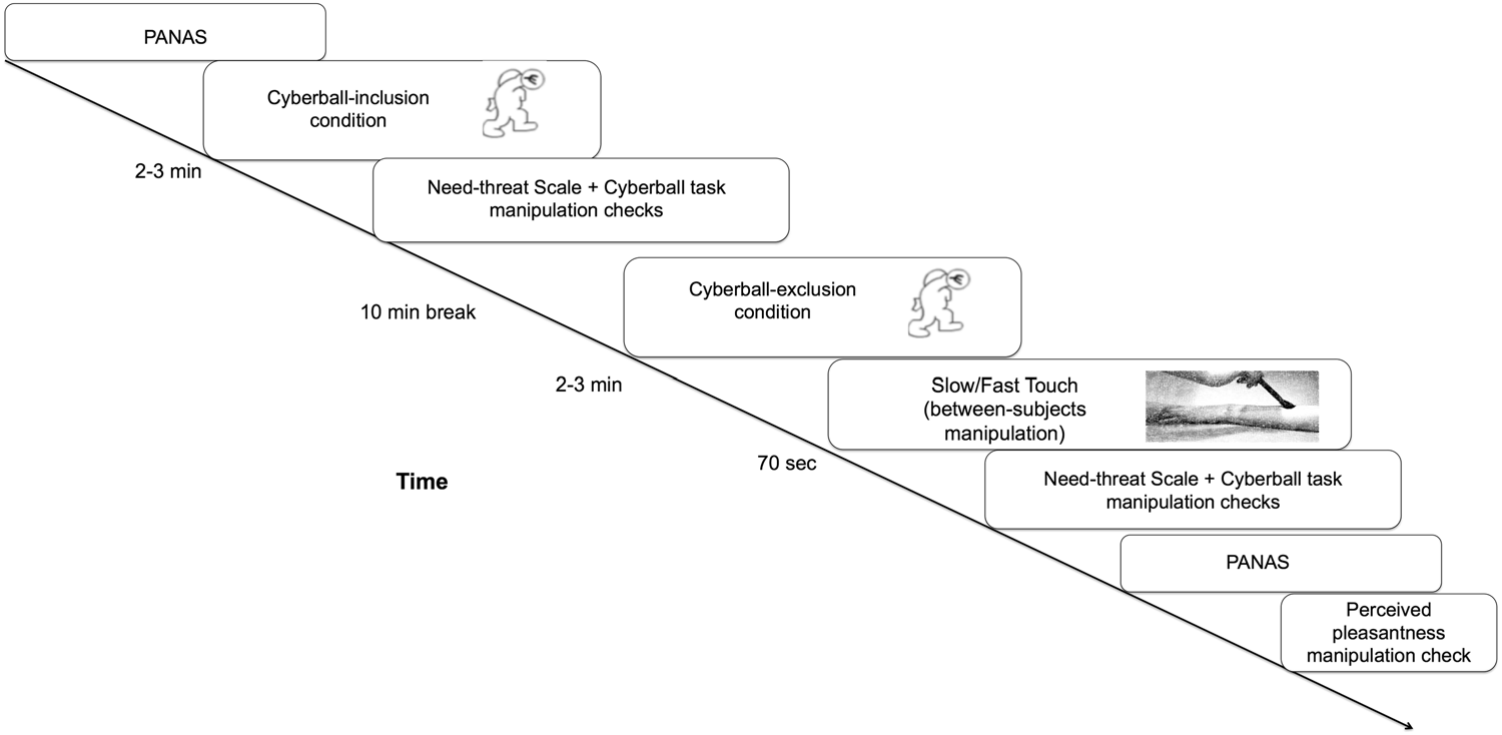
Schematic representation of the study procedure. All participants completed the same experimental procedure, in the exception of receiving slow or fast touch after the Cyberball exclusion condition, depending on their assigned group. PANAS, Positive and Negative Affect Schedule; Slow touch (3 cm/s); Fast touch (18 cm/s).

Following a 10-minute break of Sudoku-like activities, participants played the Cyberball-Exclusion game for 2–3 minutes; they received the ball 2 initial times, while they were excluded in the remaining ball-tosses. Upon completion, participants were blindfolded. The experimenter stroked the participant’s marked skin areas for 70 seconds with a soft brush (Natural hair Blush Brush, No. 7, The Boots Company) in either: CT-optimal speed (3 cm/s; slow touch group) or non CT-optimal speed (18 cm/s; fast touch group), as in previous studies by our group^42,50,51^. The experimenter was trained to deliver the touch at these two different speeds. Following tactile stimulation, participants filled out the main measure of ostracism, namely the Need-threat scale. As before, participants’ responses were averaged across each subscale to yield an averaged total index of need-threat level (Cronbach α = 0.73), with lower scores indicating greater effects of ostracism (see Fig. 1 for a schematic representation of the study procedure).

### Manipulation Checks

#### Affect

The Positive Affect and Negative Affect Schedule (PANAS^52^) was used to assess potential changes in affect as in many previous studies using the Cyberball paradigm (e.g.^53,54^). The PANAS includes two affect scales, one affect scale measures positive affect and the other one measures negative affect. This measure has twenty items in total (ten items per scale), rated on a continuous five-point scale, ranging from ‘not at all’ to ‘extremely’. Scores for positive and negative affect items were summed, separately, yielding a total score for each affect scale. Higher scores indicate high (positive or negative) affect. We collected affect ratings at the beginning of the experiment, as well as upon completion of the Need-Threat scale following the Cyberball-Exclusion game and touch manipulation (at the very end).

#### Cyberball task

Two types of manipulation checks were conducted on the Cyberball task, namely, exclusion perception and attention checks. First, upon completion of the Cyberball task and need-threat scale, participants reported whether they perceived having been ‘excluded’ and ‘ignored’ during the game in order to assess whether they had experienced the Cyberball task as intended. This manipulation measure, used in most previous studies using the Cyberball paradigms (e.g.^48,55^), is separate from the main dependent variable of interest (i.e., the need-threat scale; see^48^) as it assesses the perception of ostracism rather than feelings of ostracism associated with the Cyberball game. These manipulation check items were rated on a continuous 5-point scale, ranging from ‘not at all’ to ‘extremely’. Consistent with prior research^49^, to ensure the validity of the Cyberball task we examined whether our participants experienced the task as intended, i.e. they felt excluded in the excluded condition. Thus, the two items assessing how ignored and excluded participants felt were averaged and cases with scores 2 SD above/below the mean were excluded from main analyses. Five and one participants for the fast and slow touch group, respectively, did not meet this criteria and were excluded from further analyses. Second, participants also reported an estimate on the percentage of ball tosses they received during the Cyberball game to ensure they were paying attention. No participants were excluded on this basis.

#### Perceived Pleasantness

At the end of the experiment, we collected pleasantness ratings of slow, affective CT optimal and fast, neutral non CT optimal touch from both groups to make sure that participants perceived slow touch as more pleasant than fast touch, irrespective of their assigned group, in accordance with prior literature^37,56^. We used a soft brush (Natural hair Blush Brush, No. 7, The Boots Company) to administer 16 randomized trials of 3-second tactile stimulation at CT-optimal (3 cm/s) and non CT-optimal (18 cm/s) speeds to the participant’s previously marked forearm skin areas. Note that these CT and non-CT speeds are the same speeds administered in the touch manipulation following the exclusion condition. After each trial, participants were asked to rate the pleasantness of the touch by using a scale ranging from 0 ‘not at all pleasant’ to 100 ‘extremely pleasant’. CT optimal slow and non-CT optimal fast touch ratings were averaged separately for each participant, creating fast touch and slow touch pleasantness averaged rating scores for each participant.

### Statistical Analyses

Data exploration confirmed that our continuous variables of interest were normally distributed. Moreover, tests of normality (i.e., Kolmigorov-Smirnov, Shapiro-Wilk) were conducted on this data. In spite of data being normally distributed, the assumption of homogeneity of variance throughout grouped data was violated in the inclusion condition (*p* < 0.05 on the Levene’s test), but not the exclusion condition (*p* > 0.05 on the Levene’s test), need-threat total scores. Given that group sizes were relatively equal (ratio of the larger to smallest group being less than 1.5) and thus the *F* statistic may be robust to this assumption, parametric tests were employed and reported. Nevertheless, analyses on the need-threat total scores were also conducted by using non-parametric tests (i.e., Wilcoxon Signed Rank and Mann-Whitney U on difference scores) to make sure that these results were replicated, which in fact, yielded the same pattern of results. Statistical analyses were conducted on a final sample of seventy-eight participants (slow touch group: forty-one participants; fast touch group: thirty-seven participants). Effect sizes are presented as partial eta-squared (η^2^_partial_). A 0.01 η^2^_partial_ represents a small effect size, 0.06 η^2^_partial_ represents a medium effect size and 0.14 η^2^_partial_ represents a large effect size^57^.

### Data Availability

The datasets generated during and/or analysed during the current study are available from the corresponding author on reasonable request.

## Results

**Figure 2:**
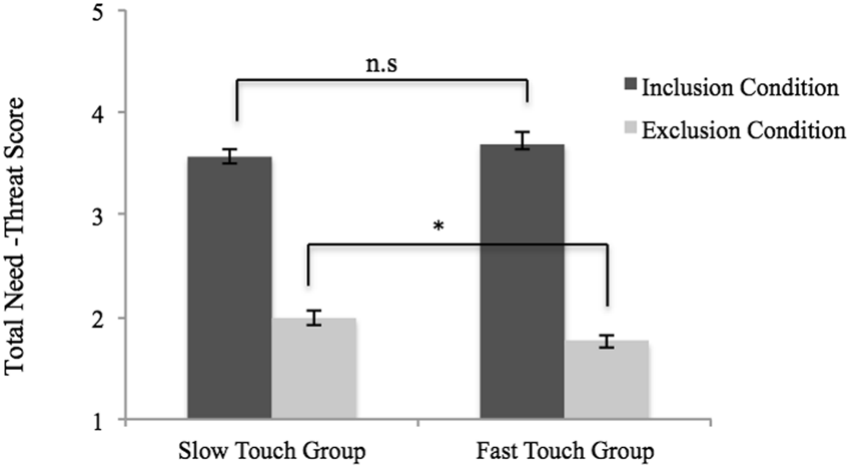
Inclusion and exclusion total need-threat score of the slow and fast touch group on a continuous 5-point scale. Lower scores indicate greater need-threat. Error bars denote ± standard error of the mean for illustration purposes. Asterisks indicate significant differences (p < 0.025) and n.s. indicate non-significant differences.

To examine the effects of slow, affective versus fast, neutral touch on ostracism, we measured participant’s need-threat level (i.e., need-threat scale; Jamieson *et al*.^48^) following the inclusion and exclusion condition and touch manipulations. Across the groups, participants reported more need-threat in the Cyberball exclusion (*M* = 1.88, *SD* = 0.41), as compared to the inclusion/baseline (*M* = 3.63, *SD* = 0.62), *F*(1,76) = 479.50, *p* < 0.001, η^2^_partial_ = 0.86. There was no effect of group, *F*(1,76) = 0.38, *p* = 0.540, η^2^_partial_ = 0.01. As predicted, the ostracism condition interacted with the group, *F*(1,76) = 4.48, *p* = 0.038, η^2^_partial_ = 0.06. Post-hoc tests, using Bonferroni adjusted alpha levels of 0.025 per test (0.05/2), showed that while threat levels between the groups did not differ at baseline (i.e., inclusion/baseline condition), *t*(63.69) = −0.81, *p* = 0.422, there was a significant group difference in the exclusion condition, following touch manipulation. Specifically, participants that received slow touch (*M* = 1.99, *SD* = 0.42) post-ostracism reported less need-threat than those that received fast touch (*M* = 1.76, *SD* = 0.37), *t*(76) = 2.46, *p* = 0.016 (see Fig. 2).

### Manipulation Checks

Across the groups, participants reported decreased positive affect (M = 23.24, SD = 7.81), but no differences in negative affect (M = 15.87, SD = 5.13), following the exclusion condition, as compared to baseline measures (positive affect: M = 29.13, SD = 7.13; negative affect: M = 15.42, SD = 5.34, respectively), *F*(1,76) = 68.17, *p* < 0.001, η^2^_partial_ = 0.47; *F*(1,76) = 0.46, *p* = 0.499, η^2^_partial_ = 0.01, respectively. Importantly, positive and negative affect did not differ by the assigned touch group of the participants, *F*(1,76) = 0.19, *p* = 0.661, η^2^_partial_ = 0.01; *F*(1,76) = 0.13, *p* = 0.715, η^2^_partial_ = 0.01, respectively; nor did touch group interact with the ostracism condition, *F*(1,76) = 0.2.42, *p* = 0.124, η^2^_partial_ = 0.03; *F*(1,76) = 1.18, *p* = 0.280, η^2^_partial_ = 0.02, respectively. Together, these results suggest that while social exclusion decreased positive affect, the type of touch received by the participants did not have a moderating effect on neither positive nor negative general affect following social exclusion.

Overall analyses conducted on the cyberball task manipulation check scores suggested that the manipulation of ostracism was effective. Participants reported having been excluded and ignored to a greater extent in the exclusion (M = 4.53, SD = 0.61) as compared to the inclusion condition (M = 1.40, SD = 0.57), *F*(1,76) = 1165.16, *p* < 0.001, η^2^_partial_ = 0.94. In addition to these manipulation checks, participants were also asked to estimate the percentage of ball-tosses that they received during the game to ensure they were paying attention to the task. As expected, participants reported receiving a lower percentage of ball-tosses during the exclusion (M = 5.59%, SD = 7.67%), as compared to the inclusion condition (M = 32.35%, SD = 8.93%), *F*(1,76) = 724.39, *p* < 0.001, η^2^_partial_ = 0.91. Furthermore, group did not interact with the ostracism condition on the ostracism and attention manipulation checks, *F*(1,76) = 0.95, *p* = 0.332, η^2^_partial_ = 0.01, *F*(1,76) = 0.02, *p* = 0.895, η^2^_partial_ = 0.00, respectively, indicating that participants perceived the cyberball games in a similar manner, irrespective of their assigned group.

Analyses conducted on the pleasantness ratings scores of both type of touch (CT and non-CT optimal touch) suggested that affective and neutral touch was perceived as expected in both groups. Participants perceived slow touch (*M* = 67.68, *SD* = 16.22) as more pleasant than fast touch (*M* = 51.07, *SD* = 13.45), *F*(1,76) = 67.69, *p* < 0.001, η^2^_partial_ = 0.47. Importantly, group did not interact with touch velocity, *F*(1,76) = 2.19, *p* = 0.143, η^2^_partial_ = 0.03, indicating that slow touch was perceived as more pleasant than fast touch, irrespective of the assigned group and hence our manipulations were successful in terms of perceived pleasantness of touch.

## Discussion

The present study investigated the effects of slow, affective touch on the subjective effects of social exclusion or, ostracism. Given the importance of social support and in particular, embodied social support^17^^,^^20^ in buffering negative experiences, we predicted that slow affective touch would lessen the distress caused by ostracism. Consistent with prior research^3,48,55^, we found that people report more distress following conditions of social exclusion. However, contrary to past research^58,59^, we found no differences in negative affect following conditions of social exclusion, possibly indicating that the mere presence of another individual providing touch, i.e., social reconnection, may attenuate the negative affect elicited by social exclusion^10,33^ (although see also^55,60^ for no effects on mood following social exclusion). Nevertheless, our main finding was that this distress was significantly lessen in a group that received slow, affective touch following the ostracism manipulation, as compared to a fast, ‘neutral’ touch group, although neither manipulations was sufficient to totally eliminate the effects of social exclusion.

The current findings supported our predictions. Slow touch (CT-optimal speed), which was perceived as more pleasant than fast touch (non-CT optimal), was able to buffer to a degree the effects of interpersonal threatening experiences such as ostracism. Moreover, we found that affective touch did not have a more general effect on improving affect post-exclusion. Instead, it appears that affective touch is particularly effective in reducing feelings of social exclusion. Whereas one can assume that many other affective modulations may reduce the effects of social exclusion, e.g. reading a happy versus a sad story, the present findings are important because the only variable manipulated was the velocity of touch between individuals. Thus, many general and cognitive factors, e.g. social proximity, social desirability, attention, general mood effects, can be excluded as candidate explanations of our effect. Instead, our finding suggests that a unique type of embodied, tactile interaction between individuals is capable of modulating the subjective effects of social exclusion. These findings are consistent with research pointing to the role of touch in the formation of social bonds^26,27,50^, as well as the findings that social exclusion can motivate interpersonal reconnection^9,10^, including touch^33^. Our findings therefore extend prior research indicating that touch leads to seeking interpersonal connection and pro-social behaviour following social exclusion^33^, and suggest that particularly this type of dynamic slow touch, which is associated with neurophysiological specificity and conveys social support^38,25,43^, buffers ostracism-related effects.

Our findings add to the growing literature on the overlap between the physical and social pain system. Recent studies have found that CT-optimal touch reduces subjective^41^ and neural responses to noxious stimulation^42^. The current study suggests that CT-optimal, affective touch affects the ‘social pain’ associated with ostracism, at least in the short run. These findings thereby support the notion that factors that influence physical pain may also modulate social pain, consistent with the physical-social pain overlap hypothesis^4^.

More generally, the present findings corroborate and extend prior research on the beneficial effects of social support, and particularly embodied social support, on threat and stressful life events. While past research suggests that social support may possess stress-protective effects on social stressors, including social exclusion and correlated activity in the dACC^16^, such studies did not directly assess the actual role of social support in buffering ostracism-related effects. Subsequently, a recent study has shown that receiving supportive text messages during social exclusion leads to increased activity in left prefrontal areas as well as changes in activity in the ventral ACC during both social exclusion and support, suggesting a possible neurocognitive regulatory mechanism underlying cognitive-emotional support^22^. Moreover, one study assessed the buffering effects of the presence of a friend (versus a stranger) on the distress caused by ostracism^21^. However, as aforementioned, these manipulations may entail many confounds. Thus, here we employed comparable conditions of embodied social support, namely slow-affective touch versus fast-neutral touch, to minimise potential familiarity and social desirability confounds^17,20,23^ (see also supplementary Figure 1 for a pilot study indicating that there were no difference of familiarity associated with the touch velocities used in this study). Embodied social support has been shown to reduce activity in brain regions implicated in emotion regulation when in threat, thereby pointing to the pivotal role of physical contact with others in how we cope with stressors^17^. Thus, our findings extend existing literature by suggesting that embodied social support may not only buffer threats to physical safety^17^, but also threats to social connection, e.g., ostracism.

To the best of our knowledge, this is the first time that a sensory-affective manipulation is shown to buffer ostracism-related effects. However, our findings should be considered in light of study limitations and directions for future research. First, we employed a mixed design, with a between-subjects manipulation of affective touch, which may thus entail potential individual variability confounds. Nevertheless, such between-subjects manipulation of touch was deemed necessary as a result of pilot studies suggesting that the Cyberball paradigm could not be optimally implemented in a repeated measures design (i.e., subjects suspected of the rejection manipulations when these were repeated). Second, we only tested women to control for gender effects related to touch^27,46^. However, it’s worth mentioning that men and women seem to differentially benefit from verbal and tactile support^15,31,61^. Thus, future studies should investigate whether the present results extend to men.

Third, although using comparable conditions of embodied social support has great methodological advantages, it may in turn raise other experimental inquiries associated with this type of social support. For instance, are these modulatory effects on ostracism mediated by bottom-up physiological mechanisms or top-down learned expectations of pleasantness and support linked with this specific type of touch? As far as support is concerned, bottom-up mechanisms reflect sensory signals input processing (in this case CT-afferent signaling in response to slow touch in CT skin), whereas top-down mechanisms reflect higher cognitive processes (including learned expectations linked to a stimuli, in this case social support to slow touch^25^). Similarly, as far as pleasantness is concerned, bottom-up mechanisms reflect sensory signals input processing (in this case CT-afferent signaling in response to slow touch in CT skin), whereas top-down mechanisms reflect higher cognitive processes pertaining to pleasure (including learned expectations linked to the valence of the tactile stimuli^38,50^) that may influence how (sensory-tactile) stimuli are experienced^43^. Consequently, affective touch experience involves a complex interplay between bottom-up and top-down processes. Future investigations examining the effects of touch at contrasting velocities (slow versus fast) in CT versus non-CT skin are needed to provide insight into the separate involvement of bottom-up CT-afferent signaling and top-down expectations of support in the face of ostracism.

Furthermore, higher order top-down processes (e.g., individual differences in attachment style and social context) may influence our perception of social support, including affective touch^43,23^, and consequently, our psychological responses to stress^62^ and even pain. Interestingly, research suggests attachment style moderates the effects of slow affective touch on noxious stimulation^42^. Concurrently, such effects depend on social contextual factors (e.g., touch by romantic partners^45^). Thus, it is possible that these top-down factors may not only modulate the effects of affective touch on physical pain but also on social pain. Future research is needed to examine potential dispositional and contextual factors at play.

Fourth, in accordance with prior research in the field (e.g.^42,45,51^), the present study employed cosmetic-like soft brushes to deliver the touch. On the one hand, using cosmetic-like soft brushes to deliver the touch, as compared to skin-to-skin contact, allowed us greater experimental control over confounding factors such as differences in skin temperature, sweating rates and uncomfortable feelings. Moreover, soft, hairy like materials are frequently used in toys and gadgets as proxies for social support and affiliation, and pet stroking studies have showed that petting, and particularly stroking^63^, hairy animals, i.e., dogs, attenuates transient physiological and psychological responses such as blood pressure, heart-rate and state anxiety^63–66^. Similarly, in monkeys, it has been shown that touch and proximity to softness, i.e., ‘contact comfort’, even if it is artificial softness as in a built-in ‘soft’ (made out of cloth) surrogate mother, is a proxy for the mammalian need for social attachment^67^. On the other hand, it remains possible that brush stroking may have missed essential mechanisms of everyday socio-tactile interactions, and related bottom-up and top-down expectations of skin-to-skin social support. Thus future research should examine whether skin-to-skin contact, as compared to human and robot-based tactile stimulation by the use of soft brushes, may elicit different responses to feelings of ostracism.

Finally, other interactive mechanisms, such as thermoregulation, could mediate the buffering effects of affective touch. Interestingly, social exclusion is associated with an experience of ‘coldness’, e.g., leads to lower room temperatures estimations while increasing desire for warm food or drinks^68^. Conversely, CT afferents responds optimally to dynamic touch around 32 °C^36,69^ and thus, affective touch in this context may also provide some kind of ‘warm’ embodied support. Indeed, mammalian physical contact with conspecifics involves social thermoregulatory processes, which rely on thermosensory and somatosensory pathways in response to slow touch^35,32^. Given the functional and anatomical proximity of C thermoregulatory and mechanosensitive C afferents, it is possible that CT afferents may mediate circuits important for thermoregulatory behaviours^32^, including social exclusion. Future research is needed to examine whether social thermoregulatory mechanisms mediate the present effects.

In sum, the present study corroborates and extends prior literature on the regulatory function of slow, affective touch. It demonstrates for the first time that slow, affective touch, as compared to fast, ‘neutral’ touch, can lessen to a certain degree the distress caused by ostracism. These findings point to the soothing function of affective touch, particularly in the context of social separation or rejection. Future research is needed to specify the neurophysiological mechanisms involved.

## Electronic supplementary material

Supplementary Material
