# Supplementary material for: The soothing function of touch: affective touch reduces feelings of social exclusion
Source: Sci Rep. 2017 Oct 18;7:13516. doi: 10.1038/s41598-017-13355-7 (PMC5647341; doi:10.1038/s41598-017-13355-7)
Supplement: Supplementary file 1 — Supplementary Material [file 41598_2017_13355_MOESM1_ESM.pdf]

Supplementary Material

THE SOOTHING FUNCTION OF TOUCH: AFFECTIVE TOUCH REDUCES FEELINGS OF SOCIAL EXCLUSION

Mariana von Mohr*[1], Louise P. Kirsch[1], Aikaterini Fotopoulou[1]

[1]Research Department of Clinical, Educational and Health Psychology, University College London, London, United Kingdom

Supplementary Material

We conducted a short pilot (n=12 females) to test potential familiarity effects associated with the touch. We tested such familiarity effects on four different speeds/velocities (i.e., very fast, 30 cm/s; fast, 18 cm/s; slow, 3 cm/s; very slow, 0.3 cm/s), as dictated by microneurography studies (e.g., Loken et al., 2009), thus including very fast and very slow speeds in addition to the speeds used in our study, to capture the inverted U-shape. We used a soft cosmetic brush (Natural hair Blush Brush, No. 7, The Boots Company; as done in our study) to administer 12 randomised trials (3 trials per speed). After each trial, participants were asked to answer the question *'How often do you think you experience this type of touch with this particular speed in your everyday life?'* using a scale ranging from 0 'not often at all' to 100 'extremely often'. To examine whether there are any differences in familiarity within these speeds, we employed a repeated-measures ANOVA, specifying the within-subjects factor of speed (very slow, 0.3 cm/s; slow, 0.3 cm/s; fast, 18 cm/s; very fast, 30 cm/s), with planned comparisons on the two touch speeds (fast, 18 cm/s versus slow, 3 cm/s) used in our study. The main effect of speed was statistically significant, $F(3,33)=15.27$, $p<.001$, but there were no differences in familiarity between fast (18 cm/s) and slow (3 cm/s) touch, $t(11)=.58$, $p=.572$. Thus, results from this pilot suggest that there is no potential familiarity confounds effects between the two speeds used in our study. However, as shown in Supplementary Figure 1, individuals seem to find less familiar the more 'extreme' speeds (i.e., very slow at .3 cm/s and very fast at 30 cm/s).

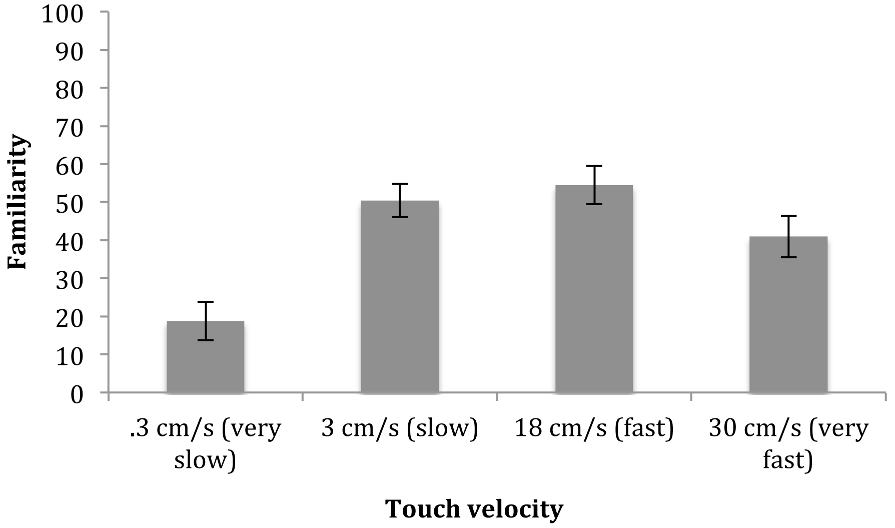

Supplementary Figure 1. Pilot data on familiarity and the velocity of the touch. Note. Error bars denote ± standard error of the mean.
